# Aging on Chip: Harnessing the Potential of Microfluidic Technologies in Aging and Rejuvenation Research

**DOI:** 10.1002/adhm.202500217

**Published:** 2025-06-12

**Authors:** Limor Zwi‐Dantsis, Vignesh Jayarajan, George M. Church, Roger D. Kamm, João Pedro de Magalhães, Emad Moeendarbary

**Affiliations:** ^1^ Department of Mechanical Engineering Roberts Building University College London London WC1E 6BT United Kingdom; ^2^ Department of Genetics Harvard Medical School Boston MA 02115 USA; ^3^ Wyss Institute for Biologically Inspired Engineering Harvard University Boston MA 02215 USA; ^4^ Department of Biological Engineering Massachusetts Institute of Technology Cambridge MA 02139 USA; ^5^ Department of Mechanical Engineering Massachusetts Institute of Technology Cambridge MA 02139 USA; ^6^ Genomics of Ageing and Rejuvenation Lab Department of Inflammation and Ageing, College of Medicine and Health University of Birmingham Birmingham B15 2WB United Kingdom; ^7^ BioRecode Ltd. London EC4N 7BE United Kingdom

**Keywords:** aging research, microfluidics, organ‐on‐a‐chip, rejuvenation

## Abstract

Aging is a complex process and the main risk factor for many common human diseases. Traditional aging research using short‐lived animal models and two‐dimensional cell cultures has led to key discoveries, but their relevance to human aging remains debatable. Microfluidics, a rapidly growing field that manipulates small volumes of fluids within microscale channels, offers new opportunities for aging research. By enabling the development of advanced three‐dimensional cellular models that closely mimic human tissues, microfluidics allows more accurate investigation of aging processes while reducing costs, resource use, and culture time. This review explores how microfluidic systems, particularly organ‐on‐chip models, can improve our understanding of aging and age‐related diseases, bridge the gap between animal models and human biology, and support the discovery of rejuvenation therapies. We highlight their role in monitoring aging biomarkers, analyzing functional cellular changes, and identifying longevity‐promoting compounds. The ability of microfluidics to detect, analyze, and remove senescent cells is also discussed, along with emerging applications such as partial reprogramming for cellular rejuvenation. Furthermore, we summarize how these devices support single‐cell analysis and recreate specific tissue microenvironments that influence aging. Insights from microfluidic approaches hold promise for developing therapeutic strategies to extend healthspan and promote longevity.

## Graphical abstract

This review highlights recent advances in microfluidic technologies for modeling human aging and age‐related diseases. It explores how organ‐on‐chip platforms improve physiological relevance, enable rejuvenation strategies, facilitate drug screening, detect senescent cells, and identify biomarkers. The potential of microfluidics to replace animal models and advance personalized aging medicine is also discussed.

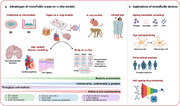

## 1 Introduction

The elderly population has continued to expand, and it is estimated that by 2050, over 1.5 billion people will be aged 65 and older.^[^ ^1^ ^]^ However, this increase in longevity often lacks a corresponding growth in health span, compromising the quality of life and leading to economic and social burdens.^[^ ^2^ ^]^ Aging stands as the major risk factor for most chronic diseases, which account for 95% of direct health costs for seniors and projecting Medicare spending in the US to exceed $1.2 trillion by 2030.^[^ ^3^ ^]^ As a result, there is a growing emphasis on unraveling the underlying mechanisms of aging and developing interventions to target the aging processes, making it a priority field of research.^[^ ^4^ ^]^

Human aging is a complex, multifactorial process driven by cumulative changes at the molecular, cellular, and tissue levels. At the molecular level, it involves genomic instability, telomere attrition, epigenetic alterations, loss of proteostasis, deregulated nutrient sensing, and mitochondrial dysfunction. These alterations lead to cellular‐level consequences such as senescence and stem cell exhaustion, ultimately manifesting in tissue‐level disruptions including impaired regeneration and altered intercellular communication.^[^ ^5^ ^]^ For a more in‐depth overview of the hallmarks of aging and their associated molecular‐to‐tissue biology, readers may refer to recently published review papers.^[^ ^6^, ^7^ ^]^

Given this complexity, modeling human aging requires experimental platforms that can recapitulate its multi‐faceted pathophysiology and clinical symptoms.^[^ ^8^ ^]^ The study of aging involves various paradigms and approaches, each with its own limitations.

In vitro models, particularly traditional 2D cell cultures,^[^ ^9^, ^10^ ^]^ have been crucial for understanding molecular aging mechanisms. They offer advantages such as cost‐effectiveness and ease of manipulation but lack the complexity of tissue‐scale and systemic cues disrupted in aging.^[^ ^11^ ^]^

Alternative models for studying aging have employed unicellular organisms such as the budding yeast *Saccharomyces cerevisiae*. Studying replicative aging in yeast has revealed insights into evolutionarily conserved enzymes and pathways regulating aging^[^ ^12^, ^13^, ^14^ ^]^ as well as potential interventions for mitigating its effects.^[^ ^15^ ^]^ However, traditional yeast lifespan analysis on agar plates and manual separation cannot track molecular markers and yeast biology differs from humans.^[^ ^16^ ^]^

Animal models, including nematodes, flies, and rodents, play a vital role in aging research due to their shorter lifespans and genetic manipulability, making them useful for mimicking human aging phenotypes.^[^ ^17^ ^]^ These models have provided many insights into the fundamental understanding of aging mechanism. However, animal models come with several limitations when applied to human aging and age‐related diseases. Key issues include limited generalizability due to species‐specific differences in disease manifestation and physiological traits. For example, animal models often exhibit physiological differences, age at different rates, and may not fully replicate human conditions like cardiovascular disease,^[^ ^18^ ^]^ immune response,^[^ ^19^ ^]^ neurodegenerative diseases,^[^ ^20^ ^]^ and drug metabolism.^[^ ^21^ ^]^ Furthermore, in vivo models, such as rodents and non‐human primates, suffer from limitations such as high costs, low throughput, ethical concerns, and physiological differences compared to humans. The use of shorter lifespan or accelerated aging models, along with the absence of long‐term longitudinal data, can further distort the natural aging process and hinder our understanding of aging in humans. Additionally, many animal models rely on inbred strains, which lack genetic diversity and may not fully represent evolutionary complexity.^[^ ^22^ ^]^

**Figure 1:**
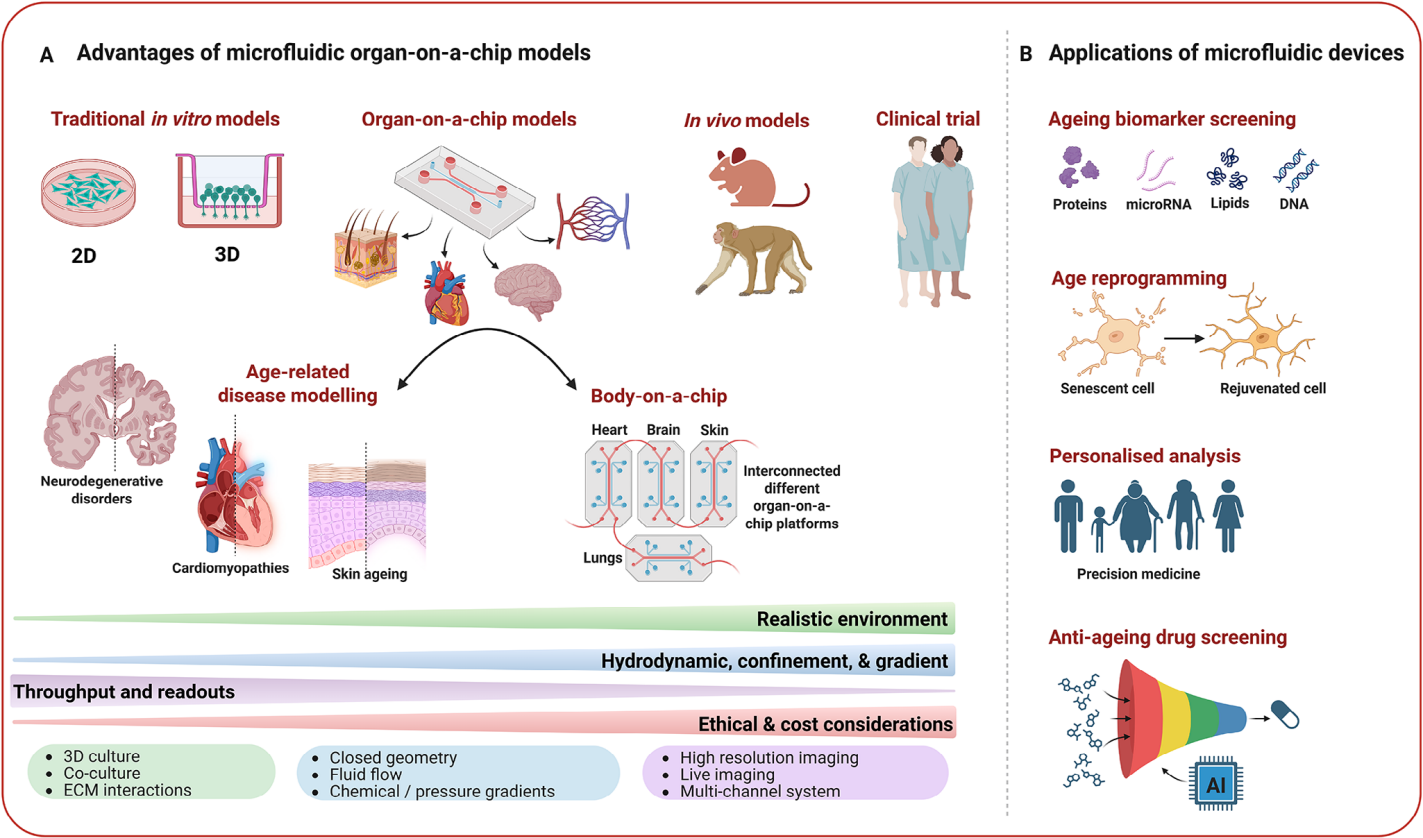
Broad application of organ‐on‐chip systems and microfluidics for breakthroughs in aging and rejuvenation. (A) The development of single‐organ‐on‐chip and multi‐organ chips enables high‐throughput experiments with minimal reagents, significantly reducing research time and costs compared to traditional in vitro and in vivo models, while more closely replicating human biological processes. These microfluidic devices serve as powerful tools for modeling diseases related to aging, including neurodegenerative disorders, skin aging, and cardiovascular diseases. (B) Furthermore, they can be used to identify new biomarkers of aging, to partially reprogram cells, and to screen for perturbations that rejuvenate the transcriptome. They also aid in designing personalized strategies for preventing and treating aging‐related diseases by using cells from donors of diverse genders (male and female), ages (young and old), or genetic backgrounds. Finally, microfluidic models play a crucial role in identifying and screening novel targets for anti‐aging interventions. Created with BioRender.com.

In recent years, microfluidics has emerged as a promising tool for studying aging, offering of physiologically relevant 3D environments with high‐throughput capabilities that surpass the limitations of traditional 2D cultures and bridge the gap between animal models and human As a multidisciplinary technology, microfluidics processes or manipulates small volumes of fluids (from pico to microliters) within channels measuring 10–1000 µm.^[^ ^23^ ^]^ Traditional fabrication methods, such as photolithography and soft lithography, particularly using polydimethylsiloxane (PDMS), remain widely used due to their cost‐effectiveness and biocompatibility. However, newer approaches, including 3D printing, injection molding, and laser micromachining, offer greater flexibility for rapid prototyping and the creation of complex architectures. Design considerations are equally critical and are tailored to the specific application, focusing on parameters such as channel geometry, fluid dynamics, material properties, and the integration of on‐chip components like valves, sensors, and actuators. A comprehensive overview of the design and fabrication of microphysiological systems is beyond the scope of this review; readers are referred to existing reviews for further detail.^[^ ^24^, ^25^, ^26^ ^]^ Microfluidic devices offer numerous advantages, including reduced resource consumption and costs, shorter culture times, and improved simulation of pathophysiological conditions in 3D cellular systems compared to other model systems (**Figure** **1**).^[^ ^27^ ^]^ Therefore, microfluidics platforms have been extensively employed in various domains of life science research, such as developmental biology, disease modeling, drug discovery, and clinical applications,^[^ ^28^ ^]^ positioning this technology as a significant avenue in the field of aging research.

This review focuses on recent implementations of microfluidic technologies to model age‐related changes and study age‐associated diseases. Additionally, we discuss how the power of microfluidics can be leveraged for rejuvenation studies and the development of therapeutic interventions.

## 2 Organ‐on‐a‐chip Modeling of Aging and Age‐Related Diseases

“Organ‐on‐a‐chip” technology employs microfluidic devices that replicate the structure and function of human organs.^[^ ^29^ ^]^ These microdevices, fabricated in diverse sizes and shapes, feature hollow channels lined with cultured cells and tissues that can be exposed to dynamic flow. Some devices provide biomechanical cues, such as shear stress, hydrostatic pressure, tensile stretch, or compression, offering precise control over physiological conditions and serving as effective tools for studying organ function, diseases, and responses to treatments.^[^ ^30^ ^]^ Recent advances in stem cell technology have enabled the integration of patient‐specific cells into microfluidic systems, creating personalized preclinical models.^[^ ^31^ ^]^ A more detailed understanding of organ‐on‐a‐chip can be found in several recent in‐depth reviews.^[^ ^25^, ^31^, ^32^ ^]^

The evolution of organ‐on‐a‐chip systems, from single‐organ chips (e.g., lung,^[^ ^33^, ^34^, ^35^ ^]^ liver,^[^ ^36^, ^37^, ^38^ ^]^ heart,^[^ ^39^, ^40^, ^41^ ^]^ kidney,^[^ ^42^, ^43^ ^]^ blood vessels^[^ ^44^, ^45^, ^46^ ^]^ and skin,^[^ ^47^ ^]^ bone,^[^ ^48^, ^49^ ^]^ thymus,^[^ ^50^ ^]^ and the lymphatic system,^[^ ^51^ ^]^ among others) to multi‐organ chips and human‐on‐a‐chip systems,^[^ ^52^ ^]^ demonstrates scientific progress and attracts attention for their advantages over traditional in vitro techniques and animal studies.

Organ‐on‐a‐chip technology can offer valuable insights into the aging process at cellular and organ levels. As an advanced tissue engineering approach, it can recreate the features of aging organs, generating tissue models that emulate key parameters related to aging and associated pathologies. This technology provides a notable opportunity to simulate the progression of aging‐related diseases in vitro, enabling the investigation of early disease events and identifying molecular signatures for potential therapeutic targets (Figure 1).

We first focus on how organ‐on‐a‐chip systems have contributed to modeling various aging‐related diseases including neurodegenerative disorders, cardiovascular diseases, and skin aging.

### 2.1 Neurodegenerative Disorders

Aging is the leading risk factor for most neurodegenerative diseases, which currently have limited treatment options. The microfluidic organ‐on‐chip approach holds promise for advancing our mechanistic understanding of the pathogenesis of neurodegenerative diseases, bringing new hope for treating such conditions.

#### 2.1.1 BBB‐on‐a‐chip

The brain is protected by the blood–brain barrier (BBB), a selectively semipermeable barrier that separates circulating blood from the brain and central nervous system (CNS). It is crucial for maintaining the brain and CNS homeostasis, as it prevents the entry of toxic substances into the brain.^[^ ^53^ ^]^ The BBB undergoes several deleterious changes during normal aging and neurodegeneration. At the molecular level, there is a downregulation of tight junction proteins, such as occludin and claudins, which weakens the BBB integrity and increases its permeability. Additionally, the upregulation of pro‐inflammatory cytokines, such as TNF‐α, IL‐1β, and IL‐6, exacerbates this dysfunction.^[^ ^54^ ^]^ At the cellular level, endothelial cells experience oxidative stress and mitochondrial dysfunction, impairing their ability to maintain homeostasis. Pericytes (PCs), which are critical for BBB function, show decreased coverage and viability, further promoting vascular leakage. Astrocytes (ACs) also become reactive, driving neuroinflammation through increased cytokine secretion.^[^ ^55^ ^]^ Structurally, the basement membrane thickens, which impairs nutrient exchange and contributes to vascular stiffness, thereby compromising transendothelial electrical resistance (TEER).^[^ ^55^ ^]^ These cumulative molecular, cellular, structural and functional changes compromise brain health, with BBB dysfunction serving as a key factor in the progression of neurodegenerative diseases such as Alzheimer's disease (AD), Parkinson's disease (PD), and multiple sclerosis.^[^ ^56^, ^57^, ^58^ ^]^

Early BBB research focused on 2D models, utilizing endothelial cells (ECs) in transwell chambers to form tight junctions, which regulate substance passage between the bloodstream and brain.^[^ ^59^ ^]^ Monoculture models, while simple and cost‐effective, lack other cellular components, exhibit low TEER, have high paracellular permeability, and poor transporter expression, making them inadequate for replicating barrier functions. To better mimic in vivo conditions, multicellular environments where ECs are co‐cultured with other brain cells such as ACs, PCs, neurons, or microglia have been developed.^[^ ^60^, ^61^, ^62^ ^]^ These co‐culture models provide higher TEER values and lower permeability than monolayer models, though they still lack some in vivo characteristics due to the absence of shear stress.^[^ ^63^ ^]^ Recent advancements have led to the development of 3D models that more accurately mimic the BBB's in vivo structure and function, providing a better platform for studying cell interactions and the regulation of BBB permeability.^[^ ^59^ ^]^ Early in vitro BBB models incorporated artificial tubes and shear stress mimicking in vivo blood flow, but lacking physiological interactions with pericytes and astrocytes.^[^ ^64^, ^65^, ^66^ ^]^ Despite their advanced features, these models faced challenges such as technical complexity, high cell load, and long setup times, limiting their scalability and applications.^[^ ^60^, ^67^ ^]^

Microfluidic‐based models more closely replicate the in vivo BBB morphology and function with thicker membranes (<10 µm) and accurately recapitulate the shear stress exerted by steady blood flow on endothelial cells to mimic normal physiological conditions.^[^ ^61^ ^]^ They have been further characterized by their reduced permeability to drugs/markers, making them more comparable to in vivo permeability studies than the traditional transwell system.^[^ ^68^, ^69^ ^]^ Griep et al. pioneered the first BBB device, featuring a compartmentalized model with a single culture of human brain endothelial cells. This system revealed that TNF‐α negatively impacts BBB integrity, a common feature in neurodegenerative diseases.^[^^]^ Park et al. reported the development of an in vitro microfluidic organ‐on‐a‐chip model of the BBB using stem cell‐derived human brain microvascular endothelium (iPS‐BMVECs) along with primary human PCs and ACs.^[^ ^71^ ^]^ This model replicated the high barrier function of the human BBB for at least one week, demonstrating low permeability and the expression of efflux pumps and transporter functions.^[^ ^71^ ^]^ In another study, Campisi et al. created a 3D human BBB microvascular network model by incorporating ECs, PCs, and ACs inside a hydrogel matrix injected into a microfluidic channel.^[^ ^72^ ^]^ The cells self‐assemble into 3D vascular architectures that replicate the in vivo neurovascular organization with BBB features offering a valuable platform for drug discovery and the quantitative assessment of the permeability of molecules of interest, thereby aiding in predicting neuro‐therapeutic transport efficacy. Subsequently, Hajal et al. provided a detailed protocol describing the steps required to fabricate this BBB model and measure physiologically relevant molecular permeabilities.^[^ ^73^ ^]^ Maoz et al. used three interconnected microfluidic chips, rather than a single chip, to model influx across the BBB, the brain parenchymal compartment, and efflux across the BBB, developing a compartmentalized neurovascular unit (NVU) model that uncovered previously unknown metabolic interactions between the brain microvasculature and neurons, demonstrating that vascular metabolites significantly increase the neuronal synthesis and secretion of neurotransmitters such as glutamate and GABA. This NVU system, comprising multiple interconnected devices that more accurately replicate the BBB, offers potential for various applications, including the assessment of drug delivery systems aimed at crossing the human BBB and the evaluation of drug penetrance, localized target effects, and off‐target effects more directly than previous BBB/NVU models thereby offering new insights into neurodegenerative diseases such as stroke, traumatic brain injury, and AD.^[^ ^74^ ^]^ Recently, Zeng et al. developed a BBB‐on‐a‐chip to model the BBB under both normal and abnormal conditions, effectively recreating its intricate microenvironment. The introduction of a triple co‐culture system incorporating ACs and microglia further enhanced cell‐cell communication within this environment, providing a platform for modeling neurodegenerative diseases.^[^ ^75^ ^]^ Other models of BBB chips have been created, demonstrating their ability to replicate a human in vivo‐like permeability barrier for modeling both healthy and diseased neurovascular systems.^[^ ^76^, ^77^, ^78^ ^]^ These chips have also been used in modeling neuroinflammation^[^ ^79^ ^]^ and studying the transport of various substances, including drugs, nanocarriers,^[^ ^80^ ^]^ and tumor extracellular vesicles,^[^ ^81^ ^]^ across the BBB.

With advancements in microfluidic technology and a deeper understanding of the anatomical/ biological features of the BBB, these models could eventually replace current 2D models, providing new insights into BBB dysfunction. To realize this potential, future BBB‐on‐chip and microfluidic models will need to be simpler in design and highly scalable. Additionally, developing an ideal microfluidic platform that can predict the behavior of drug delivery systems in the brain during the early stages of drug development would be highly beneficial, as the fidelity of BBB models plays a critical role in optimizing delivery systems—especially for brain‐directed gene therapies like adeno‐associated viruses (AAVs).^[^ ^82^ ^]^ Unlike the simpler delivery of therapies to organs such as the liver for blood‐borne proteins,^[^ ^83^ ^]^ brain‐directed therapies must efficiently cross the BBB and target nearly all affected cells within the central nervous system. Therefore, high‐fidelity microfluidic BBB models that closely mimic in vivo conditions will be instrumental in advancing the delivery of viral vector therapies for neurodegenerative diseases like Alzheimer's and Parkinson's.

#### 2.1.2 Alzheimer's Disease‐on‐a‐chip

Aging is the main risk factor for AD, the most common neurodegenerative disorder, affecting one in ten individuals aged over 65.^[^ ^84^ ^]^ AD is marked by memory loss and general cognitive decline and remains poorly understood in terms of its underlying mechanisms, with limited treatment options.^[^ ^85^, ^86^ ^]^ The hallmarks of aging, including genomic instability, epigenetic alterations, mitochondrial dysfunction, cellular senescence, and chronic inflammation, contribute to the progression of AD.^[^ ^87^ ^]^ Genomic instability and epigenetic changes disrupt neuronal function, while mitochondrial dysfunction leads to oxidative stress, exacerbating neurodegeneration. Cellular senescence and altered intercellular communication promote neuroinflammation, further impairing BBB integrity and accelerating disease progression.^[^ ^7^ ^]^ Pathologically, AD is characterized by the deposition of β‐amyloid (Aβ) peptides,^[^ ^88^ ^]^ with emerging evidence suggesting that a dysfunctional BBB is a key factor in the causes and consequences of AD.^[^ ^89^ ^]^ Cell culture models to investigate AD‐associated BBB impairment have previously been developed utilizing transwell inserts, with the external addition of Aβ peptides at high concentrations for short periods.^[^ ^90^, ^91^ ^]^ However, these models lack many features of the human brain in AD and fail to reproduce the gradual accumulation of soluble Aβ peptides.

**Figure 2:**
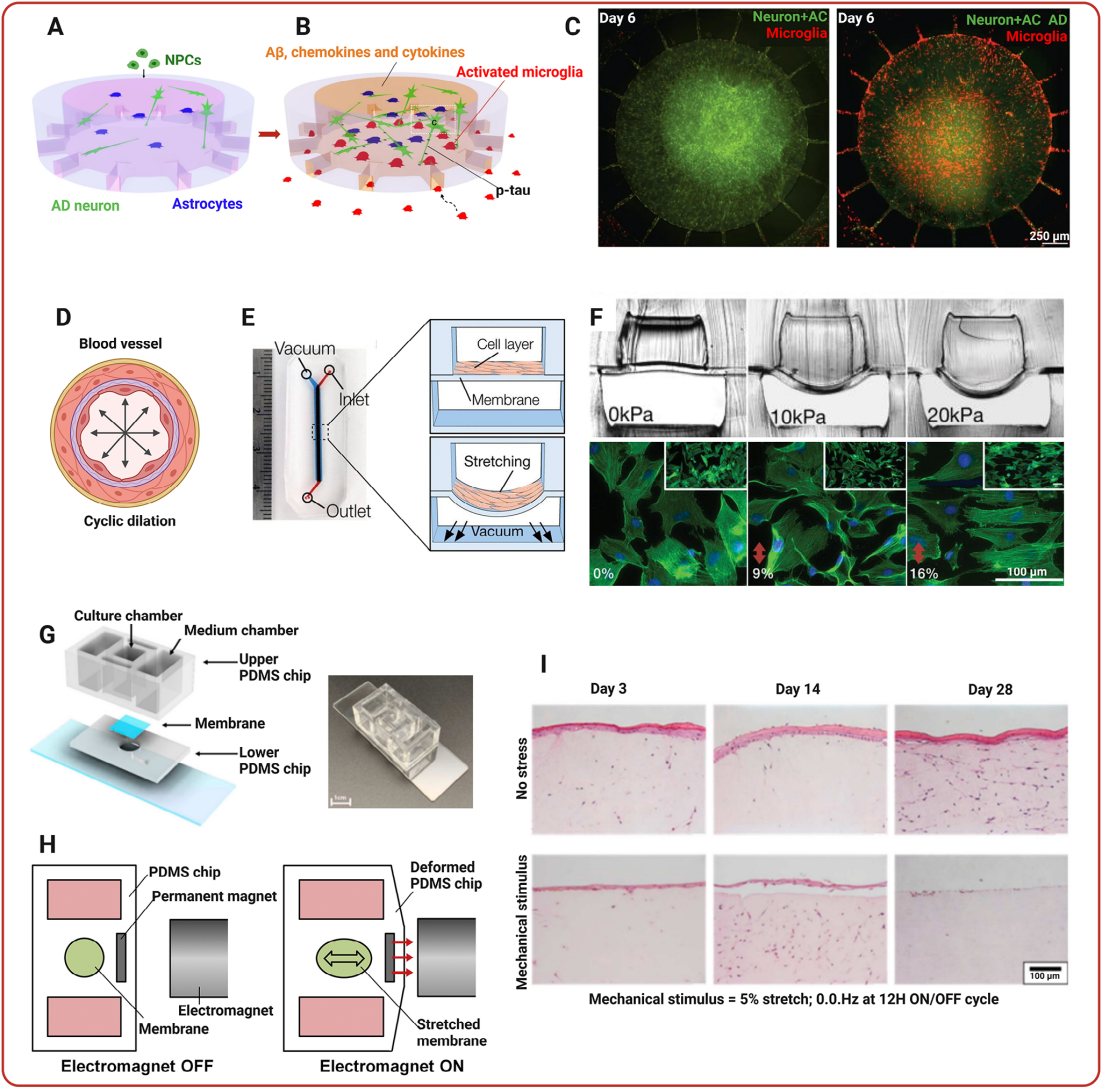
Organ‐on‐a‐chip platforms for modeling age‐related diseases. Top panel: (A) Schematic of the Alzheimer's Disease (AD) brain model, where the central chamber is loaded with AD neuron/astrocyte differentiated cells and the angular chamber contains adult microglia. Migration channels (10  ×  50  ×  500 µm, height  ×  width  ×  length) connect the chambers, forming gradients of soluble factors. (B) Illustration showing that microglia are activated by soluble factors from the AD culture in the central chamber and begin migrating. (C) Confocal images highlighting activated microglia (red) infiltrating the central chamber, which contains AD neurons/astrocytes (Neuron+AC AD, green). Adapted with permission.^[^ ^95^ ^].^ Copyright 2018, Springer Nature. Middle panel: (D) Diagram depicting the cyclic strain experienced by blood vessels due to blood flow. (E) Photograph of the progeria‐on‐a‐chip model next to a ruler, showing the media inlet/outlet and the vacuum port used to introduce cyclic strain. Cross‐sectional images of the progeria‐on‐a‐chip under varying pressure drops, illustrating membrane deformation. (F) Microscopic images of F‐actin‐stained cells (green) after 24 h under different levels of strain. The images show a clear change in cellular orientation with 16% strain. Adapted with permission.^[^ ^118^ ^].^ Copyright 2017, John Wiley and Sons Ltd. Bottom panel: (G) Schematic representation of the flexible aging skin‐on‐a‐chip structure. Image of the actual flexible aging skin‐on‐a‐chip. (H) Illustration showing how an electromagnet is used to stretch the skin. (I) Hematoxylin and eosin‐stained skin sections demonstrating differences in epidermal thickness. A 5% stretch, applied to induce aging, results in a noticeable decrease in epidermal thickness. Adapted with permission.^[^ ^119^ ^].^ Copyright 2018, Elsevier B.V. and with permission.^[^ ^120^ ^].^ Copyright 2021, MDPI.

Inadequacies of 2D cell culture in analyzing the disease along with the low predictive validity of animal‐based in vivo models^[^ ^92^ ^]^ have promoted the development of 3D in vitro microfluidic systems that exhibit key events in AD pathogenesis. These microfluidic systems, which mimic the BBB phenotype, microvascular network model,^[^ ^72^, ^73^ ^]^ and brain interstitial flow^[^ ^93^, ^94^ ^]^ have emerged as powerful tools for studying AD with precise control of 3D cellular and noncellular microenvironments. For instance, Shin et al. developed a 3D AD model using a 5‐channel microfluidic device to mimic the BBB.^[^ ^56^ ^]^ This model integrates human neural progenitor cells with familial AD mutations and a tube‐shaped brain endothelial cell (bEC) barrier, replicating key vascular changes seen in AD, such as increased BBB permeability and decreased expression of tight and adherent junction proteins. The study successfully recapitulated AD pathology and BBB dysfunction, highlighting increased permeability, reduced junction protein expression, elevated ROS, MMP‐2, and inflammatory cytokines like interferon‐gamma (IFNγ).^[^ ^56^ ^]^ Ko et al. advanced this work by developing a microfluidic platform that allows direct cell‐cell interactions and replication of the BBB environment.^[^ ^92^ ^]^ They developed a device with a 3D self‐assembled BBB vascular network (consisting of ECs, ACs, and PCs) alongside neuronal microtissues in a single system. This integration achieved better outcomes, such as closer to physiological vessel diameters, brain‐like permeabilities  and fully perfusable vasculature.^[^ ^92^ ^]^ Park et al. presented a new 3D human AD triculture model on a microfluidic platform by incorporating neurons, ACs, and microglia. This model is designed to study key features of AD, including Aβ aggregation, phosphorylated tau accumulation, and neuroinflammatory activity, aiming to provide a more precise human brain model for studying neural‐glial interactions and drug discovery in AD (Figure 2).^[^ ^95^ ^]^ Furthermore, to study the molecular mechanisms underlying immune cell infiltration in AD, Jorfi et al. created a 3D human neuroimmune axis model using a microfluidic chip that supports the culture of stem cell‐derived neurons, ACs, and microglia, along with peripheral immune cells. This model showed a significant increase in T cell infiltration in AD cultures, with CD8+ T cell infiltration leading to microglial activation, exacerbating neuroinflammation and neurodegeneration. Blocking the CXCL10–CXCR3 axis effectively prevented T cell infiltration and neurodegeneration in AD cultures, identifying this chemokine as a potential therapeutic approach.^[^ ^96^ ^]^ Other studies focused on unraveling the effects of tau proteins, commonly referred to as neurofibrillary tangles (NFTs), and their role in the progression of AD.^[^ ^97^, ^98^ ^]^

Recent developments using patient iPSC‐derived neuronal tissues have successfully discriminated between samples from Alzheimer's patients and age‐matched controls following differentiation, providing a valuable tool for identifying disease‐specific changes and potential therapeutic targets.^[^ ^99^ ^]^ Building on these findings, the difference in the rate of differentiation of these cells, combined with the capability of the microfluidic BBB environment, offers hope for exploring AD mechanisms and preventative strategies. To further enhance AD model accuracy, continuous re‐circulation of the media could be incorporated into the microfluidic devices. This would enable better factor distribution and prevent the depletion of secreted factors. Further improvement could be achieved by controlling physiological flow levels, which would reduce permeability and improve model fidelity. Another challenge to overcome is extending the co‐culture duration to generate disease‐specific phenotypes using neuronal microtissues, which requires several weeks or even months. Despite these challenges, microfluidic systems remain valuable for studying the BBB's role in AD and can be adapted to incorporate patient‐specific brain organoids or neurospheres for personalized investigations. Additionally, these systems offer a platform for moderate‐throughput drug screening aimed at inhibiting BBB dysfunction or enhancing BBB integrity.

#### 2.1.3 Parkinson's‐Disease‐on‐a‐chip

PD is a progressive neurodegenerative disorder and the second most frequent after AD.^[^ ^100^, ^101^ ^]^ The risk of developing PD increases with age, and the majority of individuals diagnosed with PD are typically older adults. This age‐related risk is closely linked to the hallmarks of aging, such as mitochondrial dysfunction, oxidative stress, and neuroinflammation, which play a crucial role in PD progression. Mitochondrial dysfunction leads to impaired energy production and increased neuronal vulnerability, while oxidative stress accelerates dopaminergic neuron loss in the substantia nigra. Chronic neuroinflammation, driven by activated microglia and astrocytes, exacerbates neuronal damage and disrupts cellular homeostasis. Additionally, impaired protein homeostasis contributes to the accumulation of α‐synuclein aggregates, a key pathological feature of PD.^[^ ^102^ ^]^ The disease is apparent through symptoms such as slow movements, difficulty in walking, tremors, and rigidity. This process may activate microglia, causing neuroinflammation and the release of reactive oxygen species. Despite extensive research, the exact mechanism of PD progression remains poorly understood, and as a result, there is currently no cure. The limited availability of effective treatments is attributed to a lack of comprehensive models that accurately represent PD's complex in vivo tissue and cellular conditions. Microfluidic devices offer a potential platform for replicating the dynamics of both the neurological and cellular components in an in vitro model of PD.^[^ ^103^ ^]^ These devices provide a controlled and precise environment, allowing to mimic certain aspects of the intricate interactions between neurons, glial cells, and the pathological accumulation of α‐synuclein aggregates observed in PD. Fernandes et al. developed a microfluidic device featuring two separate chambers where human H4 neuroglioma cells and n9 microglial cells were cultured, aiming to investigate their cell‐to‐cell interactions and biochemical communication.^[^ ^104^ ^]^ A similar microfluidic setup was employed to explore the transport of mitochondria along dopaminergic axons isolated from mice.^[^ ^105^ ^]^ Seidi et al. utilized a microfluidic platform to study the impact of neurotoxin 6‐OHDA on pheochromocytoma (PC12) neuronal cells, establishing an in vitro model of PD.^[^ ^106^ ^]^ This approach has contributed to developing compact and economically viable microfluidic devices suitable for moderate through‐put screening.^[^ ^107^ ^]^ Spitz et al. developed a multi‐sensor integrated organ‐on‐a‐chip platform that monitors electrophysiological, respiratory, and dopaminergic activity of human midbrain organoids derived from a PD patient and a healthy individual. Their study demonstrated that microfluidic cultivation reduced necrotic core formation and improved tissue differentiation and the replication of essential PD pathological parameters. Additionally, significant rescue effects were reported when using 2‐hydroxypropyl β‐cyclodextrin (HP‐β‐CD), highlighting the platform's potential for drug screening and personalized medicine applications.^[^ ^108^ ^]^ In another study, de Rus Jacquet created a 3D human BBB chip to explore astrocyte role in BBB weakening in PD. They found that ACs from female donors with the PD‐related LRRK2 G2019S mutation are pro‐inflammatory and do not support functional capillary formation in vitro. Inhibiting MEK1/2 signaling was shown to reduce the inflammatory profile of these mutant ACs and restore BBB formation, shedding light on mechanisms regulating barrier integrity in PD. The vascular changes observed in the BBB chips are also present in the substantia nigra of PD patients, indicating that ACs may play a significant role in PD‐associated dysfunction.^[^ ^109^ ^]^ Other models incorporating micro bioreactors and human induced pluripotent stem cell (iPSC)‐derived human neural stem cells (hNESCs) from both healthy individuals and PD patients have been established. These models have proven effective in the development of personalized therapies.^[^ ^76^, ^110^ ^]^

Microfluidic systems for Parkinson's disease models show promise but still face challenges such as replicating the brain's complex environment, maintaining long‐term culture stability, and efficiently differentiating dopaminergic neurons, which could be addressed by advanced co‐culture systems, optimized nutrient delivery, refined differentiation protocols, and precise non‐invasive sensing strategies.

#### 2.1.4 ALS‐Disease‐on‐a‐chip

Another age‐associated neurodegenerative disease is Amyotrophic Lateral Sclerosis (ALS), a fatal neurodegenerative disorder affecting upper and lower motor neurons.^[^ ^1^ ^]^ Several hallmarks of aging, including genomic instability, mitochondrial dysfunction, and cellular senescence, contribute to ALS pathology. Genomic instability leads to DNA damage accumulation, impairing neuronal function. Mitochondrial dysfunction results in oxidative stress, exacerbating motor neuron degeneration. Cellular senescence and chronic inflammation further accelerate neurodegeneration by disrupting intercellular communication and promoting toxic microenvironments.^[^ ^111^ ^]^

Various studies have aimed to replicate the loss of neuromuscular junctions (NMJs) observed in ALS using microfluidic platforms. Uzel et al. co‐cultivated motor neurons derived from mouse embryonic stem cells (ESCs) and myoblast‐derived muscle strips within a 3D hydrogel matrix, successfully demonstrating NMJ formation.^[^ ^112^ ^]^ Ozaki et al. co‐cultured human ESC‐derived motor neuron (MN) spheroids with an endothelial cell (EC) barrier in microfluidic devices, revealing that bidirectional signaling between the neural and vascular networks aided in the formation of synaptic junctions and neuronal function^[^ ^113^ ^]^ The same group developed an ALS‐on‐a‐chip device by co‐culturing skeletal muscle bundles and human iPSC‐derived motor neurons into a spheroid, providing insights into ALS pathogenesis and highlighting the importance of autophagy and degradation of TAR DNA binding protein–43 (TDP‐43) in the MNs.^[^ ^114^ ^]^ Machado et al. developed an open microdevice where mouse embryonic stem cell‐derived motor neurons and ACs grow through microchannels, forming functional NMJs with contractile myofibers in a separate compartment. In a motor neuron disease model, motor neurons cocultured with ALS‐related *SOD1^G93A^* ACs showed denervation and reduced myofiber contraction. This phenotype was improved upon treatment with the kinase inhibitor necrostatin. This platform can be adapted for modeling other neuromuscular diseases; however, the authors used mixed animal cell sources, limiting clinical relevance.^[^ ^115^ ^]^ In another study, human iPSCs were differentiated into spinal neural progenitor cells for a spinal cord‐on‐chip system, exhibiting increased neuronal activity and enhanced expression of neuronal‐specific genes compared to a 2D in vitro model. Including iPSC‐derived endothelial cells further increased neuronal activity and induced vascular‐neural interaction genes, offering insights into endogenous signaling and potential disease treatment.^[^ ^116^ ^]^

Many existing microfluidic models focus primarily on motor neurons, often overlooking the growing recognition that ALS impacts both motor neurons and muscle tissue. This gap limits the physiological relevance of these models. Therefore, increased use of models integrating co‐cultures of motor neurons and muscle cells could significantly enhance model accuracy for ALS. Another shortcoming is the limited capacity of current systems to mimic the dynamic blood flow found in vivo. Advances in microfluidic design, such as improved vascular channel networks and more sophisticated cell integration, would help address these challenges, making ALS models more comprehensive and reflective of the complex disease environment.

To summarize, the field of microfluidic models for neurodegenerative diseases has significantly progressed over the last few years. Nevertheless, future microfluidic models should focus on additional hallmarks of aging, many of which are associated with neurodegenerative diseases. Among these hallmarks are immunosenescence, which leads to increased inflammation and a diminished response to senescent cells, further aggravating neurodegenerative conditions; a decline in autophagy and the proteasome system with age, resulting in protein misfolding, characteristic of many neurodegenerative diseases; and mitochondrial dysfunction, marked by decreased energy production and increased oxidative stress, which also plays a critical role in these diseases.^[^ ^117^ ^]^ Other advancements should focus on improving the interface between microfluidic flow and in vitro vasculature, particularly when working with larger organoids or organ models. A more robust system that can integrate shear stress and physiological differences, such as blood versus culture media, will provide better insights into the real‐world performance of drug delivery mechanisms.

### 2.2 Cardiovascular Diseases

Numerous studies highlight that aging leads to a decline in cardiac function and an upsurge in myocardial apoptosis in normal individuals.^[^ ^121^, ^122^ ^]^ Hallmark features of aging in cardiovascular diseases include endothelial dysfunction, chronic low‐grade inflammation, oxidative stress, and increased vascular stiffness. These changes contribute to impaired blood vessel function, reduced regenerative capacity, and a heightened risk of conditions such as atherosclerosis, hypertension, and heart failure. Additionally, age‐related alterations in cardiac structure and function, such as myocardial fibrosis and impaired mitochondrial function, further exacerbate disease progression.^[^ ^123^ ^]^ Given the escalating global elderly population and the associated surge in cardiovascular‐related mortality, there is a pressing need for models and screening platforms specifically addressing age‐related cardiac diseases.

The introduction of microfluidic chips has revolutionized in vitro heart tissue studies by enabling advanced on‐chip models for cardiac ischemia, fibrosis, and cardiotoxicity in a more realistic 3D environment than traditional 2D plating architectures. The heart‐on‐a‐chip system, incorporating cyclic stretch,^[^ ^124^ ^]^ electric impulses,^[^ ^125^ ^]^ anisotropic organization,^[^ ^126^ ^]^ and fluid flow,^[^ ^127^ ^]^ has proven superior for pharmaceutical analysis and biomedical applications. Recently, the growing use of 3D printing and direct laser writing photolithography in microfluidics has shown enhanced potential in creating endothelialized myocardial tissue and understanding the heart's mechanical properties ex vivo.^[^ ^128^, ^129^, ^130^ ^]^

Notably, Budhathoki et al. developed an aging cardiac tissue chip model to study aging‐mediated cardiovascular disease. The model, recapitulating an infarcted aging heart through hemodynamic loading, demonstrated decreased cardiac function, and increased myocardial apoptosis, characteristic of aging cardiomyopathy. Additionally, their analysis revealed a higher prevalence of senescent cells, marked by enlarged and flattened nuclei, along with an increased expression of senescence markers and reactive oxygen species (ROS).^[^ ^131^ ^]^

In other studies, a chip model was employed to investigate Hutchinson‐Gilford progeria syndrome (HGPS), a rare disorder characterized by accelerated aging and early onset of atherosclerosis, leading to cardiovascular diseases.^[^ ^132^ ^]^ Ribas et al. developed progeria‐on‐a‐chip model to examine the impact of biomechanical strain on healthy and HGPS‐induced pluripotent stem cell‐derived smooth muscle cells (iPSC‐SMCs) in the context of vascular aging and disease. Physiological strain induced a contractile phenotype in primary SMCs, while pathological strain mimicked the hypertensive phenotype observed with angiotensin II treatment. HGPS iPSC‐SMCs exhibited an intensified inflammatory response to strain, marked by elevated inflammation markers and DNA damage (**Figure** **2**).^[^ ^118^ ^]^ Atchison et al. developed 3D arteriole‐scale vessel‐on‐chip models with endothelial and smooth muscle cells derived from both healthy individuals and Progeria patients' iPSCs.^[^ ^133^, ^134^ ^]^ These Progeria vessels exhibited increased inflammatory cytokines and diminished vasoactivity compared to controls, highlighting the vascular endothelium's role in Progeria.

Microfluidic systems for modeling cardiovascular disease in aging face several limitations, including replicating the complex multicellular environment of aged cardiovascular tissues, maintaining the long‐term viability of aged cells, and accurately simulating age‐related mechanical and biochemical cues. Additionally, these models often struggle with incorporating the dynamic nature of blood flow and pressure changes seen in aging vasculature. Future microfluidic models could involve developing advanced co‐culture systems of various cell types found in aged cardiovascular tissues, optimizing long‐term culture conditions to sustain aged cells, and integrating microenvironmental controls to mimic age‐related changes in mechanical stress and biochemical signals. Furthermore, incorporating dynamic flow systems that can simulate age‐related hemodynamic changes would enhance the physiological relevance of these models, providing better insights into cardiovascular aging and disease progression.

### 2.3 Skin Aging

In addition to the age‐related diseases discussed above, skin aging is another complex biological process influenced by both intrinsic factors, such as metabolic processes, and extrinsic factors, such as environmental exposures leading to structural and functional damage. Key hallmark features include oxidative stress, resulting from an imbalance between reactive oxygen species and antioxidant defences, and chronic low‐grade inflammation that accelerates tissue degradation. Cellular senescence plays a central role by impairing regeneration and promoting the secretion of pro‐aging factors. Mitochondrial dysfunction further compromises energy production and cellular homeostasis.^[^ ^135^ ^]^ Signs of aging include fine wrinkles, loss of elasticity, impaired wound healing, age spots, and changes in pigmentation and the extracellular matrix.^[^ ^136^ ^]^ Understanding the various factors contributing to skin aging is essential for developing effective skincare approaches. While numerous studies have explored skin aging induced by ultraviolet light (UV), limited research has focused on aging caused by physical or chemical stimuli due to ethical and safety concerns. Therefore, there is a crucial need for an in vitro model to conduct experiments in these domains.

Recent advancements in 3D skin cell culture techniques, such as 3D bioprinting,^[^ ^137^, ^138^, ^139^ ^]^ organoids,^[^ ^140^ ^]^ and organs‐on‐chips, offer promising avenues.^[^ ^141^ ^]^ Organ‐on‐a‐chip technology, in particular, stands out for its ability to recreate 3D aspects of skin with simplicity and cost‐effectiveness.^[^ ^142^ ^]^ More recently, several human‐aged skin equivalents have been reported. Lim et al. created aged skin equivalents, or wrinkled skin‐on‐a‐chip (WSOC), by subjecting co‐cultured human fibroblasts and keratinocytes to uniaxial stretching. The skin equivalents were perfused with media and stretched for up to 7 days at 0.01 or 0.05 Hz frequencies. Results revealed increased wrinkling at 0.01 Hz, while at 0.05 Hz, the stratum corneum nearly disappeared, indicating harm to the epidermis. Collagen and related protein production decreased in stretched equivalents, suggesting dermal damage. The WSOC model proves valuable for studying skin aging, providing insights into the effects of repeated tensile stress (Figure 2).^[^ ^119^ ^]^ Jeong et al. developed a flexible skin‐on‐a‐chip (FSOC), utilizing human fibroblasts and keratinocytes. This aging model aimed to accelerate the aging process in a full‐thickness skin equivalent by applying periodic mechanical stimulation mimicking circadian rhythms over 28 days. The accelerated aging was evident through reductions in epidermal layer thickness, contraction rate, and Myb secretion. Markers of aging, including increased β‐galactosidase gene expression, reactive oxygen species, and transforming growth factor‐β1 secretion, were observed (Figure 2).^[^ ^120^ ^]^ In other studies, Kim et al. utilized a ‘pumpless skin‐on‐a‐chip’ device to mimic human skin's structural and functional responses. They used this model to explore the anti‐aging properties of Curcuma longa leaf extract (CLLE, a natural product cosmetic ingredient)^[^ ^143^ ^]^ and coenzyme Q10 (an anti‐aging agent and antioxidant).^[^ ^144^ ^]^ The skin equivalent, comprising rat tail collagen, human dermal fibroblasts, and epidermal keratinocytes, was cultured in a polydimethylsiloxane (PDMS) chip with a central cylindrical chamber and side chambers for medium circulation. A porous membrane separated the skin substitute from the channel. This pumpless skin‐on‐a‐chip employed a gravity flow system to rotate the medium through the microfluidic channel. Drug testing with CLLE demonstrated enhanced skin barrier function in the epidermis, indicating potential applications in cosmetics, pharmaceuticals, and clinical studies, offering an animal‐free alternative.^[^ ^143^ ^]^ Increased concentrations of Q10 led to higher cell density, increased epidermal layer thickness, elevated filaggrin expression, and an increase in TGF β‐1 secretion.^[^ ^144^ ^]^

Skin‐on‐a‐chip models hold great promise beyond cosmetics and pharmaceuticals, potentially serving in clinical settings as alternatives to animal studies, with future 3D aging skin models incorporating immune cells, capillaries, nerve cells, hair follicles, and sweat glands.

### 2.4 Multi‐Organ Systems

Aging is a systemic degenerative process that affects multiple tissues and organs. Some aging‐related diseases are also mediated by inter‐organ crosstalk and interactions.^[^ ^145^ ^]^ While most single‐organ‐on‐a‐chip models lack a systemic dimension and cross‐organ communication, advanced multi‐organ body‐on‐chip systems offer a promising approach to exploring multi‐organ physiology and whole‐body drug responses. These systems employ fluidic coupling in culture chamber bioreactors within multiwell setups or coupling of single‐organ‐on‐a‐chip units.^[^ ^146^, ^147^, ^148^ ^]^ Other systems involve fluidically linking micro‐organ chambers in the same plate, each containing different tissue types.^[^ ^149^, ^150^, ^151^, ^152^ ^]^ Configuration adjustments can be made based on the number of organs to be coupled. Additionally, more recent innovations include body‐on‐chip systems that perfuse medium through channels lined by endothelium, enhancing the replication of vascular perfusion and transendothelial transport of drugs and metabolites in the human body.^[^ ^74^, ^149^, ^153^, ^154^ ^]^ Ronaldson‐Bouchard et al. reported a tissue‐chip system linking matured human heart, liver, bone, and skin tissue through recirculating vascular flow, recapitulating interdependent organ functions.^[^ ^155^ ^]^ The interlinked tissues maintained their phenotypes over four weeks, replicated the pharmacokinetic and pharmacodynamic profiles of doxorubicin, identified early miRNA biomarkers of cardiotoxicity, and improved predictive values compared to isolated tissues or those linked without endothelial barriers. This study suggests that the developed InterOrgan tissue chip can serve as a patient‐specific model for developing therapeutic regimens and drug toxicity biomarkers, paving the way for studying aged tissues and age‐related diseases. However, limitations include the exclusion of other critical organs (kidney, gut, fat) and the need for validation with different drugs and conditions. Future improvements should incorporate these critical organs and enhance scalability and standardization for better control and quantitative readouts.

As technology advances, multi‐organ models on a single chip promise to enhance our understanding of interconnected impact of aging in different organs. These models can simulate the physiological conditions of aging organs, helping to identify interlinked biomarkers of aging, test anti‐aging compounds, and develop personalized treatments for age‐related conditions, ultimately improving health outcomes for the elderly.

## 3 Patient‐Derived Microfluidic Chips for Aging and Rejuvenation

Incorporating patient‐specific samples and compounds from donors of different ages into microfluidic platforms can greatly benefit aging research. Single‐organ chips and multi‐organ chip systems can model human aging pathophysiology and drug responses in diverse subpopulations, including gender (women versus men), age groups (young versus old individuals), genetic background or other comorbidities (i.e., chronic disorders). These models pave the way for discovering new aging biomarkers, predicting adverse outcomes, and designing personalized approaches for preventing and treating aging‐related diseases and geriatric syndromes (Figure 1).

**Figure 3:**
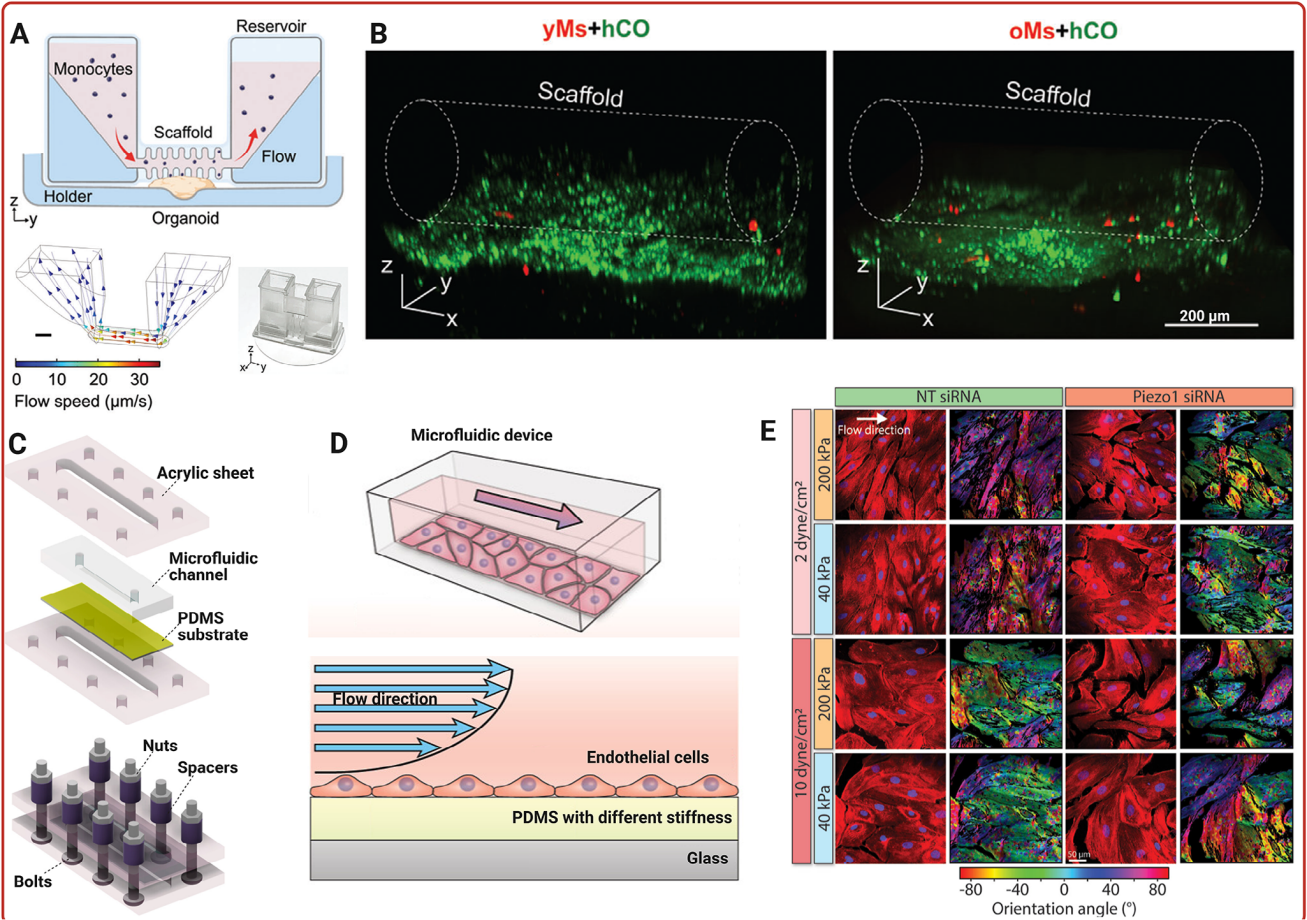
Unravellng aging mechanisms through patient‐derived organ‐on‐a‐chip platforms. Top panel: (A) A human brain organoid platform designed to study immune‐driven brain aging by analyzing the interaction between primary monocytes and human cortical organoids. The system includes a tubular, perfusable scaffold on an organoid holder that connects two reservoirs, allowing media and monocytes to flow when the platform is rocked at a 15° angle. A simulated 3D distribution of flow profiles within the device, modelled at a rocking frequency of 0.1 rpm. Next to the simulation image is a photograph of the actual microfluidic platform. (B) Confocal images illustrating the infiltration capacity of monocytes (red) from young (yMs) or old (oMs) donors into the cortical organoids (hCO). Adapted with permission.^[^ ^156^ ^].^ Copyright 2022, Wiley‐VCH GmbH. Bottom panel: (C) A 3D microphysiological platform with tunable substrate stiffness to study vasculature biomechanics during aging. (D) Schematic showing endothelial cells in microfluidic device and the possibility to expose the cells to different shear stress and substrate thickness. (E) Images of human aortic endothelial cells showing stress fibers (red) and dominant orientation angle (heat map) with or without Piezo1 knockdown under different shear stress (2 or 10 dyn/cm^2^) and substrate stiffness (200 or 40 kPa). Adapted with permission.^[^ ^157^ ^].^ Copyright 2023, American Chemical Society.

Ao et al. used a microfluidic platform to form a pancake‐shaped human brain organoid with the perfusion of immune cells that allowing to model immune‐driven brain aging using primary human monocytes from old and young donors (**Figure** **3**).^[^ ^156^ ^]^ The non‐invasive incorporation of immune cells into organoids facilitated the investigation of primary immune–organoid interaction on‐chip, replicating key features and processes of neuroimmune interaction in the aged brain with compromised BBB. Notably, the study suggested that aged monocytes could induce an aging‐related phenotype within human brain organoids without external genetic manipulation, as evidenced by increased expression of p16 and p21 within the organoids. Furthermore, neurons surrounding infiltrated aged monocytes exhibited an apoptotic morphology, supporting the hypothesis that an aged peripheral immune system may contribute to brain aging and, in severe cases, neural degeneration.^[^ ^156^ ^]^ Lai et al. investigated how aging‐associated vascular stiffening influenced endothelial cell function. Using a microfluidic organ‐on‐a‐chip model that mimicked arterial stiffness, the study examined how different stiffness levels and shear stresses affected endothelial cells' morphology, senescence, proliferation, and inflammation. The study highlighted the critical role of Piezo1, a mechanosensitive ion channel, in regulating these processes. Piezo1 was found to mediate endothelial adaptation to both protective and damaging shear stress levels, with a significant impact on inflammation and cellular senescence. Targeting Piezo1 was suggested to have therapeutic implications for age‐related vascular dysfunctions (Figure 3).^[^ ^157^ ^]^

Giza et al. developed a microfluidic system to model muscle aging and explore the cellular mechanisms underlying sarcopenia, age‐related progressive loss of muscle mass and strength. The microfluidic chip contained contractile bioengineered myobundles derived from muscle biopsies of both young, athletic, and older, sedentary adults. These myobundles were subjected to electrical stimulation through electrodes embedded along the media channel. Key transcriptional activators promoting muscle‐specific gene transcription were upregulated, indicating that primary muscle cells in the microfluidic device can proliferate and differentiate as expected. Additionally, electrical stimulation induced the expression of insulin growth factor 1 (IGF‐1) and suppressed myostatin inb young‐derived myobundles. However, these responses were attenuated in old‐derived myobundles, highlighting the variable hypertrophic response and the balance between pro‐ and anti‐atrophy gene expression in the older cohort.^[^ ^158^ ^]^ Bersini et al. used a microfluidic 3D model to investigate vascular dysfunctions associated with age‐related diseases. They employed induced vascular endothelial cells (iVECs) and smooth muscle cells (iSMCs) derived from fibroblasts of different ages and Hutchinson‐Gilford Progeria Syndrome (HGPS) patients. The study observed increased vascular permeability in iVECs from older donors, suggesting cell‐cell junction damage. Additionally, iSMCs from older donors increased endothelial barrier leakiness, while those from young donors exhibited enhanced migratory behavior. Importantly, HGPS iSMCs significantly increased vascular leakiness compared to young donors, providing insights into potential mechanisms of vascular barrier dysfunction in HGPS.^[^ ^159^ ^]^

## 4 Drug Testing for Aging Interventions and Rejuvenation

The global aging population is driving the development of new therapeutic drugs and recognising aging as modifiable, the field of “longevity pharmacology” is rapidly expanding,^[^ ^160^ ^]^ with many companies focusing on longevity therapies. Consequently, increasing number of clinical trials are testing drugs targeting age‐related diseases, offering insights into potential lifespan‐extending treatments.^[^ ^160^ ^]^

Most commercial and traditional drug screening methods rely on 2D cell cultures, which lack the complexity needed to accurately mimic the tissue microenvironment, resulting in limited predictability for drug discovery.^[^ ^161^ ^]^ Moreover, animal models such as rodents and non‐human primates, are costly, face ethical constraint and may not accurately reflect drug effectiveness and side effects.^[^ ^162^ ^]^ Furthermore, these models may not always be applicable to the genetically diverse human population in clinical settings,^[^ ^163^ ^]^ highlighting the need for more efficient, reproducible drug screening models that ensure human efficacy.

3D human cell culture, which facilitates cell‐cell and cell‐matrix interactions, has recently been applied in drug discovery for its improved prediction of in vivo responses. Microfluidics‐based 3D platforms offer superior alternatives for high‐throughput drug screening thanks to their miniaturization, low sample use, integration, and compatibility with various analytical strategies.^[^ ^164^ ^]^ These platforms, utilizing human cells to create in vitro biomimetics of the human body, open new avenues for predicting more accurate drug effects in humans.^[^ ^165^ ^]^ Moreover, microfluidic systems offer an ideal platform for precisely controlling drug concentration gradients, facilitating the analysis of cellular phenotypes in response to chemical and physical stimuli from the extracellular microenvironment. This capability allows real‐time monitoring of multiple parameters after drug administration.^[^ ^166^, ^167^ ^]^ Notably, lab‐on‐a‐chip technology has recently been recognised by the United States Food and Drug Administration (FDA) for testing in the pharmaceutical drug safety industry.^[^ ^168^, ^169^ ^]^

To date, microfluidic devices have demonstrated their usefulness for toxicity assays, pharmacological tests, and drug screening on various age‐related diseases, including neurological disorders^[^ ^106^, ^170^, ^171^, ^172^, ^173^, ^174^ ^]^ and cardiovascular diseases.^[^ ^130^, ^175^, ^176^, ^177^, ^178^ ^]^ Nevertheless, there is an increasing demand to expand the drug‐discovery pipeline of rejuvenation components. Understanding the biological causes of aging^[^ ^179^ ^]^ has opened avenues to test anti‐aging therapies using organs‐on‐chips, focusing on parameters such as telomere length, division speed, and senescent cell build‐up. Microfluidic platforms may play a crucial role in the search for new drugs against aging and age‐related diseases. Future efforts will focus on identifying therapeutic molecules that can slow the aging process and developing better aging biomarkers. With the integration of advanced computer‐based methods, including bioinformatics and AI, the design of more robust molecules and drugs is now possible. These AI‐designed compounds can then be tested for efficacy and safety in microfluidic organ‐on‐chip models, offering rapid validation and assessment before clinical trials, thereby accelerating the drug discovery process (Figure 1).

## 5 Microfluidic Approaches for Screening Age‐Associated Biomarkers Pathologies

One challenge in human aging research lies in the absence of a comprehensive set of biomarkers for effectively targeting and measuring therapeutic interventions. DNA methylation has emerged as a promising biomarker, exhibiting predictable changes over chronological time.^[^ ^180^, ^181^ ^]^ Other established biomarkers, including IgG N‐glycans,^[^ ^182^ ^]^ β‐galactosidase,^[^ ^183^ ^]^ and PSMD8,^[^ ^184^ ^]^ are commonly used to monitor aging, each reflecting specific aspects of cellular aging. Additionally, monitoring epidermal thickness and protein expression provides further insights.^[^ ^184^ ^]^ The integration of microfluidics into healthcare has revolutionised the field, offering rapid, sensitive, and portable methodologies for biomarker detection.^[^ ^185^, ^186^ ^]^ Microfluidic devices present efficient, miniaturised systems for complex analytical tasks and quick identification of disease‐specific biomarkers, making them particularly advantageous for on‐chip point‐of‐care diagnostics and real‐time condition monitoring^[^ ^187^, ^188^ ^]^ (Figure 1).

Microfluidic techniques for neurodegenerative biomarkers have mainly focused on detecting soluble proteins associated with AD, such as tau protein, amyloid‐beta or alpha‐synuclein for PD. These technologies include electrochemical immunosensors,^[^ ^189^ ^]^ quartz crystal microbalance,^[^ ^190^ ^]^ interdigitated microelectrode sensors^[^ ^191^ ^]^ and cell culture chambers.^[^ ^104^ ^]^ Fazeli et al. presented a highly sensitive microfluidic immunoassay for measuring glial fibrillary acidic protein (GFAP) in blood, addressing the need for accurate and early diagnosis of neurodegenerative disorders. GFAP, a type‐III intermediate filament, is a cell‐specific marker distinguishing ACs during CNS development.^[^ ^192^ ^]^ Increased GFAP expression has been associated with astrocytic responses to oxidative stress related to aging or hormonal mechanisms linked to the aging process.^[^ ^193^ ^]^ The developed microfluidic assay was utilized to analyze GFAP concentrations in AD, multiple sclerosis (MS), and control patients, establishing correlations with a commercial GFAP kit. AD patients displayed a significant elevation in GFAP levels compared to controls of advanced age. In contrast, individuals with MS did not exhibit a notable increase in GFAP levels compared to age‐matched controls.^[^ ^192^ ^]^ Overall, this microfluidic immunoassay offers enhanced clinical utility for GFAP detection in neurodegenerative diseases.

Additionally, microfluidic technology offers an alternative to traditional genomic and proteomic techniques in detecting skin aging biomarkers. For example, Schulze et al. employed an optical stretcher, an optical trap that captures and deforms suspended cells using two opposing laser beams, in a microfluidic setting to investigate age‐related changes in the mechanical properties of dermal fibroblasts, focusing on 14 donors aged 27 to 80. The authors reported a significant 60% increase in cellular stiffness with aging and identified alterations in cytoskeletal polymers, such as an elevation in filamentous F‐actin among older individuals.^[^ ^194^ ^]^ The observed changes in cell proliferation, motility, and actin organization imply potential consequences for detecting age‐related skin elasticity and delayed wound repair.

Recently, exosomes have emerged as early biomarkers for age‐associated pathologies, especially neurodegenerative diseases.^[^ ^195^, ^196^ ^]^ Their ability to cross the BBB has made them a potential source of attractive age‐related biomarkers. Exosomes may also carry biomarkers such as tau protein, alpha‐synuclein, and amyloid‐beta, providing a potential avenue for early diagnosis. Studies have demonstrated the presence of neurotoxic proteins in exosomes isolated from the brains, cerebrospinal fluid, and blood of patients with neurodegenerative disorders.^[^ ^197^, ^198^, ^199^, ^200^ ^]^

Microfluidic devices have been developed to detect exosomes derived from patients' samples. For example, Sina et al. reported the use of a surface plasmon resonance (SPR) platform for detecting exosomes purified from cell media and patients' serum. The SPR chip's surface was made from gold and functionalised with biotinylated anti‐CD9 or CD63 to capture exosomes.^[^ ^201^ ^]^ Ibsen et al. repurposed an alternating current electrokinetic (ACE) microarray chip device to isolate glioblastoma exosomes from undiluted plasma. This device utilises dielectrophoresis for exosome separation and fluorescence for verification, demonstrating its effectiveness in isolating exosomes and their mRNA cargo.^[^ ^202^ ^]^ Ramshani et al. presented an amplification‐free, single‐step quantification of exosome microRNAs (miRNAs) from untreated plasma samples. The microfluidic platform achieves absolute quantification of both free‐floating miRNAs and exosomal‐miRNAs with high sensitivity, offering a rapid and efficient screening tool for detecting and profiling exosome content.^[^ ^203^ ^]^ The integration of microfluidic screening technologies and exosome capturing, combined with the profiling of exosomal cargo from neural cells, could contribute to the early detection of age‐related neurodegenerative disorders, providing a more objective and timely diagnosis.

## 6 Reprogramming: Emerging Strategies for Rejuvenating Aging Cells and Tissues

To tackle the global issue of aging, innovative, safe, and effective rejuvenation treatments are essential for reversing age‐related changes and enhancing human healthspan. Cellular reprogramming and transcriptome‐based screening for rejuvenation factors are emerging as new avenues for developing such therapies (Figure 1).

Cellular reprogramming, achieved through the induction of Yamanaka factors (Oct4, Sox2, Klf4, and c‐Myc; collectively known as OSKM), has been proven to not only reprogram aged somatic cells into induced pluripotent stem cells (iPSCs)^[^ ^204^, ^205^ ^]^ but also to reverse various aging characteristics.^[^ ^206^ ^]^ Notably, recent studies have shown that transient induction of OSKM, also referred to as partial reprogramming, can mitigate age‐related symptoms and revert aged cells to a younger state without completing the full reprogramming cycle, both in vitro^[^ ^207^, ^208^, ^209^ ^]^ and in vivo.^[^ ^210^, ^211^, ^212^, ^213^ ^]^ However, despite OSKM induction's promising potential for reversing aging, it poses a risk of teratogenicity due to the loss of cell identity and the acquisition of stem‐like potential.^[^ ^214^, ^215^, ^216^, ^217^ ^]^ Additionally, its effectiveness may vary across different tissues.^[^ ^218^ ^]^

Recent findings have shown that the delivery of only three transcription factors (OSK, without the oncogene c‐Myc) has substantially increased the safety and efficacy of partial reprogramming, while reversing age‐related changes. Long‐term in vivo studies (up to 21 months) demonstrated that either local^[^ ^212^, ^219^ ^]^ or systemic^[^ ^220^ ^]^ expression of doxycycline‐inducible OSK did not result in tumor formation or adverse health changes. These results indicate that in vivo epigenetic reprogramming holds great therapeutic potential to develop interventions for age‐related diseases.

Transcriptomic reprogramming presents another approach for age reversal, which involves screening for perturbations that rejuvenate the transcriptome and shift gene regulatory networks toward a younger state.^[^ ^221^ ^]^ This strategy, distinct from cellular reprogramming, can be applied to any cell type, whether somatic or stem cell, to establish a new, stable gene regulatory network state.^[^ ^222^ ^]^ Through transcriptomics‐based screening of age‐stratified human tissues, two Hsp90 inhibitors, monorden and tanespimycin, have been identified as effective in extending lifespan and enhancing health in C. elegans.^[^ ^223^ ^]^ More recently, Plesa et al. have demonstrated that SRSF1 significantly reverses aging at the cellular level by rejuvenating the transcriptome, evidenced by improvements in senescence markers, proteasome function, collagen production, and oxidative stress management. Notably, systemic overexpression of SRSF1 achieves these effects without adverse outcomes, marking it as a possible solution for age reversal. Furthermore, SRSF1 has been shown to enhance wound healing in aged mice and is associated with increased longevity, highlighting its potential in cellular rejuvenation strategies.^[^ ^224^ ^]^

Originally, microfluidics served as a method for delivering vectors into human fibroblasts, starting with using mechanical deformation for OSKM protein delivery.^[^ ^225^ ^]^ This method evolved to include DNA delivery via ultrashort laser pulses for transient membrane disruption.^[^ ^226^ ^]^ A significant advancement in 2016 leveraged microfluidics for both vector delivery and as a reprogramming environment, achieving an efficiency 50 times higher than traditional methods. This increase was due to the accumulation of beneficial cell‐secreted factors in the confined space, drastically reducing costs and materials by about 100 times compared to conventional culture methods.^[^ ^227^ ^]^ This optimized approach led to the production of human naive iPSCs under modified conditions, showcasing the potential of microfluidics in cellular reprogramming.^[^ ^228^, ^229^ ^]^

Although the use of microfluidics for partial reprogramming and transcriptomic reprogramming is still in its early stages, this platform holds significant potential to advance the field of reprogramming and promote the rejuvenation of aging cells. For instance, microfluidic devices can provide a platform for facilitating long‐term human cell culture, enabling detailed assessments of the effectiveness and safety of long‐term partial reprogramming.^[^ ^230^, ^231^ ^]^ These long‐term studies will allow scientists to determine whether partially reprogrammed cells maintain a stable rejuvenated phenotype or if they quickly revert to their original aging state, helping to define the boundaries of a “safe” rejuvenation window. Moreover, microfluidics can be used to explore the molecular effects of reprogramming across different tissues and organs, providing a tool to assess its impact on various cell types, including post‐mitotic terminally differentiated cells like neurons, cardiomyocytes, and adipocytes, as well as on non‐dividing cells such as those in quiescent or senescent states. Another application of microfluidics is in ex‐vivo therapy, which could enable reprogramming of more complex cells outside the body, such as T cells and hematopoietic stem cells before reintroduction into their native environment. Microfluidic devices can also facilitate the use of high‐throughput screens on a chip to discover new transcriptomic reprogramming factors aimed at cellular rejuvenation. Glutamatergic neurons, neuronal stem cells, and oligodendrocytes represent a compelling target for such studies, especially for reversing age‐related cognitive decline.^[^ ^221^ ^]^ These efforts will thereby contribute to developing new rejuvenation therapies for aging and other complex conditions.

## 7 Microfluidics for Single‐Cell Analysis in Aging

**Figure 4:**
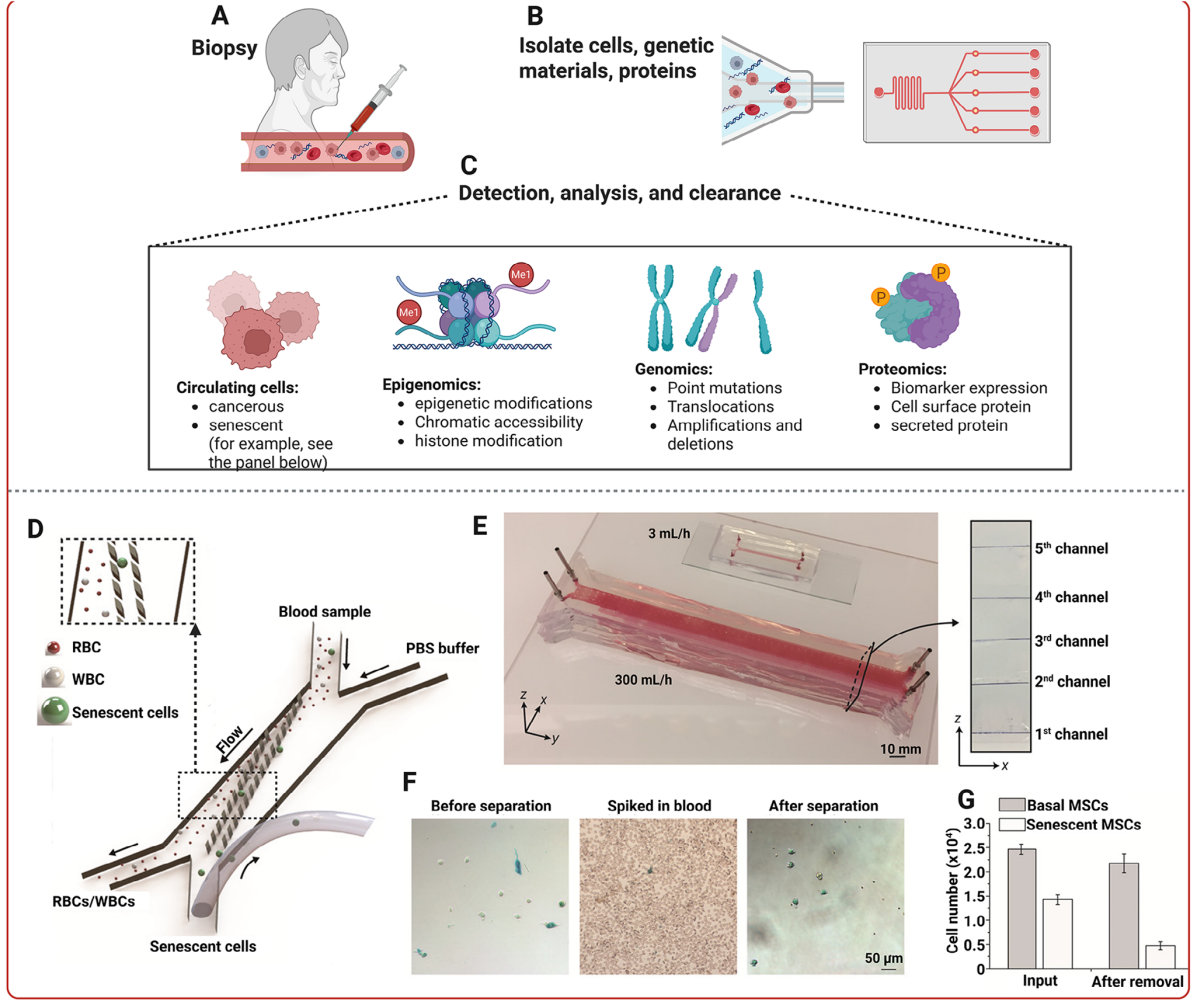
Precision aging medicine: Detection, analysis, and clearance at single cell resolution. (A‐C) Microfluidics enables the isolation of single cells from a biopsy containing mixed population, facilitating advanced omics analyses that will enhance our understanding of the molecular mechanisms underlying aging. These devices also hold tremendous potential for the detection and elimination of senescent cells, as well as for their use in 3D chip models to study their impact on the aging process. (D) A diagram illustrating how senescent cells can be filtered from blood flow using a 13‐µm 3D‐filter senescence chip. The inset shows a senescent cell being captured by the 3D filter. (E) A standard‐size senescence chip, with a flow rate of 3 mL/hour, could be scaled up and stacked to increase throughput to 300 mL/hour. (F) Mesenchymal stromal cells (MSCs), containing both senescent and non‐senescent cells, were added to human whole blood. Images were captured before and after the cells passed through the high‐throughput separation device. (G) A significant proportion of senescent MSCs (70%) were removed, with minimal loss of non‐senescent MSCs (basal MSCs). (D‐G) Adapted with permission. ^[^ ^242^ ^].^ Copyright 2018, Anatomical Society and John Wiley and Sons Ltd. Created with BioRender.com.

The traditional analysis of bulk cell populations, which averages data from millions of cells, can overlook the unique characteristics and microenvironments within those cells. Over the past decade, several types of single‐cell analyses have emerged, including single‐cell genomics, transcriptomics, proteomics, metabolomics, and epigenomics. The development of these techniques has enabled the systematic study of complex biological processes at a higher resolution, effectively probing the diversity of cellular populations.^[^ ^232^ ^]^ Microfluidic devices are particularly effective for single‐cell separation, isolating individual cells in droplets or chambers for detailed analysis.^[^ ^233^, ^234^ ^]^ Unlike conventional methods, microfluidic platforms improve accuracy and efficiency due to their ability to handle cells on a scale as small as individual cells. Furthermore, microfluidic single‐cell analysis offers higher throughput, smaller sample volumes, automatic sample processing, and a lower risk of contamination.^[^ ^235^ ^]^ Integrating microfluidic platforms with advanced techniques like high‐resolution and real‐time microscopy, as well as high‐sensitivity sensors, enhances the monitoring and analysis of cell dynamics at the single‐cell level (**Figure** **4**). For example, the combination of dynamic live‐cell imaging with microfluidic platforms can offer detailed insights into cellular behavior under controlled environments, both physically (single‐cell trapping)^[^ ^236^ ^]^ and chemically (such as in drug testing).^[^ ^237^ ^]^

Single‐cell analysis is becoming increasingly important in aging research, as it provides comprehensive insights into the cellular heterogeneity and complexity associated with the aging process. Advancements in microfluidics have been crucial for studying the dynamics of aging cells in Saccharomyces cerevisiae, a simple unicellular yeast model with a short lifespan that makes it ideal for single‐cell observation and full‐lifespan tracking. Multiple research groups have developed microfluidic devices that allow the separation of mother and daughter cells during aging and the observation of cell morphology and the expression of individual proteins throughout a cell's life.^[^ ^238^ ^]^ Research in yeast has shed light on the basic biology of aging and revealed evolutionarily conserved pathways involved in this process. Nonetheless, while this technology enables the monitoring of hundreds of cells from various strain backgrounds or media conditions weekly, its effectiveness is limited by the need for manual data analysis and the challenges involved in constructing and designing these microfluidic devices.

A lab‐on‐a‐chip approach has also been used to analyze brain cells at the single‐cell level. Kim et al. developed a neuro‐optical microfluidic platform that enables optical manipulation to study the injury and subsequent regeneration of individual mammalian axons. This approach includes observing degeneration, regrowth, and functional activities following the cutting of single axons. The study demonstrated a better understanding of the changing physiological activities and the detrimental effects of aging at a single‐cell level.^[^ ^239^ ^]^ Further integration of advanced technologies such as single‐cell RNA sequencing, single‐cell omics methods, and cell‐type‐specific genetic manipulation could significantly enhance aging research.^[^ ^240^ ^]^ These techniques, especially when combined with microfluidic models, will enhance our ability to analyze senescent cell populations and epigenetic changes, thereby further contributing to the study of aging and associated diseases at a single‐cell level.^[^ ^241^ ^]^

## 8 Microfluidics for the Detection, Analysis, and Removal of Senescent Cells

The tools of microfluidics in isolating and analyzing single cells can be leveraged for the detection and elimination of senescent cells (Figure 4). Cellular senescence, characterized by permanent cell‐cycle arrest, is a fundamental mechanism linked to aging‐related pathologies and disease.^[^ ^179^ ^]^ Senescent cells accumulate during aging, contributing to tissue dysfunction through the senescence‐associated secretory phenotype (SASP), involving the secretion of growth factors, proteases, and inflammatory cytokines.^[^ ^243^, ^244^ ^]^ Senescence is triggered by various stimuli, including DNA damage, oncogene activation, and telomere shortening.^[^ ^243^ ^]^ While senescence has beneficial effects in processes like wound healing and tumor suppression, it becomes harmful during aging, leading to proinflammatory responses.^[^ ^245^ ^]^

Detection of cellular senescence is critical for evaluating the quality of cell therapy products^[^ ^246^ ^]^ and plays a key role in enhancing our understanding of aging, cancer, and various age‐related diseases.^[^ ^247^, ^248^ ^]^ However, existing methods for detecting senescent cells, such as histochemical staining of acidic lysosomal β‐galactosidase^[^ ^249^, ^250^ ^]^ or measuring expression levels of senescence‐associated markers (p16, p21, p53) by real‐time polymerase chain reaction (RT‐qPCR),^[^ ^249^, ^251^, ^252^ ^]^ are laborious, destructive, and lack quantitative tools for analyzing heterogeneous cell populations. Therefore, there is a pressing need to develop high‐throughput, miniaturised devices for detecting senescent cells. Thamarath et al. introduced a live‐cell assay, utilizing microscale magnetic resonance relaxometry (µMRR) coupled with microfluidic devices to measure the T2 relaxation time related to intracellular iron accumulation—a potential marker for cellular senescence. Mesenchymal stromal cells (MSCs) were sorted by using a spiral microfluidic device into three size groups: unsorted, small (11–15 µm), medium (15–22 µm), and large (22–26 µm). Large MSCs exhibited senescent characteristics, including lower T2 values, limited growth potential, and extended doubling times compared to their smaller counterparts. The elevated expression of senescence markers (p16 and p21) and a high β‐galactosidase Sn Index in larger cells supported these findings.^[^ ^253^ ^]^ In another study, Lin et al. developed label‐free microfluidic cytometry for particle sizing and senescent cell identification. The system achieved an 88% accuracy rate in classifying senescent and normal human fibroblasts.^[^ ^253^ ^]^ These non‐destructive methods hold promise for quality control in cell therapy manufacturing and have broader applications in detecting and analyzing cellular senescence in various cells and tissues.

The clearance of senescent cells has emerged as a promising strategy in the field of anti‐aging and age‐related disease research.^[^ ^254^, ^255^, ^256^ ^]^ Traditional methods targeting senescence pathways, such as small molecules or protein drugs, may introduce undesired human side effects.^[^ ^257^ ^]^ To address this, Chen et al. introduced a microfluidic chip designed for ultrahigh‐throughput isolation and removal of senescent mesenchymal stem cells (MSCs) from biofluids.^[^ ^242^ ^]^ The senescence chip demonstrated successful isolation of senescent cells from both whole blood and mouse bone marrow with minimal cell damage by exploiting the increase in cell size during senescence. Operating at 300 mL/h, the high‐throughput senescence chip further emphasized its efficiency in removing senescent cells from large volumes of undiluted human whole blood. Further development could transform this senescence chip into a “senescence dialysis” system, analogous to kidney dialysis. The platform can also be extended to various cell types and senescence inducers, promising broad applications in cell enrichment, isolation, and removal of senescent cells based on changes in cell sizes to advance anti‐aging therapy.

Additionally, it has been proposed that senescent cells alter their microenvironment, modify tissues and organ functions, and produce age‐related phenotypes.^[^ ^252^, ^258^, ^259^ ^]^ Therefore, some studies have focused on generating a 3D tissue model‐on‐a‐chip to study the effects of human senescent cells on various aging‐related diseases. For example, Budhathoki et al. developed a senescent cardiac tissue chip model by inducing cellular senescence in H9c2 myoblasts through low‐dose doxorubicin treatment. The resulting senescent cells were then utilized to engineer cardiac tissue. The research revealed that the acute low‐dose doxorubicin treatment successfully induced senescence, as demonstrated by morphological and molecular markers such as enlarged nuclei, DNA damage response foci, and elevated expression of cell cycle inhibitors like p16INK4a, p53, and ROS. Under normal hemodynamic conditions, the engineered cardiac tissues displayed organized cell alignment and maintained typical cardiac cell characteristics. When exposed to hypoxia‐induced myocardial infarction, the senescent cardiac tissue model accurately replicated key disease hallmarks, including an increase in cell death and an upregulation of ANP and BNP expression.^[^ ^131^ ^]^

In another study, Pauty et al. developed a 3D dermis‐on‐a‐chip model comprising a blood vessel embedded in a collagen gel with either young or senescent skin fibroblasts. Senescent fibroblasts were observed to exert greater traction stress, suggesting increased RhoA/ROCK pathway activity. This excessive stress led to significant morphogenic rearrangement of the extracellular matrix (ECM), hinting at a potential role in the visual aspects of aged skin. Additionally, senescent fibroblasts induced angiogenesis without additional factors, with the mechanical stress contributing to sustaining angiogenic sprouts.^[^ ^260^ ^]^

These studies highlight the efficacy of the senescent tissue chip model in mimicking important aspects of aging‐related diseases, providing insights into the impact of induced senescence on cellular and molecular responses. Going forward, these senescent models can facilitate the study of SASP inhibitors, a class of drugs with implications for aging and cancer research.

## 9 Smart Microfluidics: Artificial Intelligence's Role in Shaping the Future of Aging Medicine

**Figure 5:**
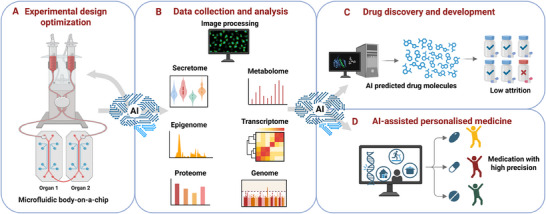
AI Meets Microfluidics: accelerating the future of aging research. Illustration of how artificial intelligence (AI) integrates with microfluidic platforms to transform aging research. (A) AI can optimize microfluidic design and experimental conditions, enhancing the precision and efficiency of aging studies. (B) AI processes high‐dimensional data from microfluidic chips to identify aging biomarkers and predict cellular behavior. (C) AI‐driven drug discovery accelerates the identification of potential anti‐aging therapies by predicting drug efficacy and toxicity in microfluidic models. (D) By integrating multi‐omics data, AI constructs personalized models of aging, paving the way for tailored therapeutic interventions. Created with BioRender.com.

The integration of artificial intelligence (AI) with microfluidic technologies is expected to significantly advance aging research (**Figure** **5**). AI, particularly machine learning, will enable the efficient processing and analysis of complex datasets generated by microfluidic systems, allowing for the rapid identification of aging biomarkers and early signs of cellular senescence that are difficult to detect through conventional methods. AI will also play a critical role in enhancing drug discovery by predicting the efficacy and toxicity of compounds within microfluidic models of aging tissues.

Through AI‐driven high‐throughput screening, researchers will be able to identify potential anti‐aging interventions more efficiently. Furthermore, AI will support the development of personalized medicine by analyzing individual genetic and phenotypic data, enabling tailored therapeutic approaches that address variability in aging across populations. Additionally, AI will enhance the design and experimental processes within microfluidic platforms. Computational models will be used to optimize fluid dynamics, tissue interactions, and experimental setups, reducing time‐consuming trial‐and‐error methods. AI systems will also automate experimental controls, ensuring optimal conditions for tissue culture and long‐term cell maintenance.

By integrating multi‐omics data from organ‐on‐a‐chip systems, AI will facilitate systems biology approaches to aging, constructing comprehensive models that elucidate molecular changes over time. This holistic approach will be essential for uncovering the complexities of aging and identifying novel therapeutic targets. However, the full potential of AI in this field will depend on overcoming challenges such as data quality, algorithm transparency, and addressing ethical considerations related to privacy. As AI technologies continue to advance, their application within microfluidic systems will provide a powerful tool for improving our understanding of aging, accelerating drug discovery, and ultimately extending health span and improving the quality of life for the aging population.

## 10 Conclusion and Perspectives

Aging is a complex process that leads to increased cellular disorder and a gradual decline in organ and tissue function, significantly contributing to human morbidity and mortality. This review highlights advances in microfluidic platforms as tools for studying and ultimately mitigating aging processes. Microfluidics have been widely used to develop organ‐on‐a‐chip models, representing a significant leap forward in translational research and precision medicine by moving away from animal models toward more accurate human‐based systems. These models have emerged to provide valuable insights into age‐related diseases, aging biomarkers, personalized treatment strategies, and facilitate the detection and analysis of senescent cells, marking a shift toward targeted interventions in aging biology and rejuvenation. Furthermore, microfluidics technology offers a more efficient and cost‐effective platform for reprogramming and exploring of new rejuvenation therapies compared to 2D cultures and animal models. This technology enables high‐throughput screening of transcriptomic reprogramming factors and presents a promising avenue for studying the long‐term effects of partial reprogramming, thereby paving the way for the development of innovative treatments to combat aging. Additionally, microfluidics systems enhance these approaches by providing precise, efficient platforms for single‐cell analysis, dissecting the cellular heterogeneity associated with aging. Finally, the integration of AI will further accelerate these advancements by enabling real‐time data analysis and optimization of experimental conditions, thus streamlining research and therapeutic development.

Microfluidics has made significant strides in biomedical research, and its applications are now beginning to translate into clinical practice through the development of point‐of‐care testing (POCT) devices and other innovative diagnostic tools. Recent legislative changes, such as the FDA Modernization Act 2.0, support the shift away from traditional animal models, enabling the use of advanced microfluidic‐based systems, including organ‐on‐a‐chip models, for preclinical testing. These models offer greater accuracy in replicating human physiology, particularly for age‐related diseases, addressing a critical need for predictive, human‐relevant data in drug development. As standardization improves and pharmaceutical companies increasingly incorporate microfluidic data into regulatory submissions, the potential for microfluidic technologies in clinical settings continues to grow, promising transformative impacts on diagnostics, personalized therapies, and ultimately, healthier aging.

Currently, some advanced applications of microfluidic devices include single‐cell analysis in simple models such as yeast, where microfluidic platforms enable high‐throughput, automated tracking of replicative lifespan and cellular responses.^[^ ^261^ ^]^ Organ‐on‐chip models for cardiac and skin tissues are also well developed, supporting drug toxicity and efficacy testing, and are increasingly integrated into pharmaceutical pipelines.^[^ ^262^ ^]^ Additionally, microfluidic devices for detecting aging biomarkers—such as proteins, exosomes, and DNA methylation—are established and suitable for both research and clinical use.^[^ ^262^ ^]^

In contrast, the most challenging applications involve modeling complex, multi‐organ interactions and chronic neurodegenerative diseases like Alzheimer's and Parkinson's. These challenges arise from the need to accurately replicate the dynamic, systemic nature of human aging, maintain long‐term viability of aged tissues, and integrate immune and vascular components.^[^ ^263^, ^264^ ^]^ Scalability, standardization, and the ability to support long‐term culture and disease progression remain significant hurdles, especially for multi‐organ and brain‐on‐chip systems.^[^ ^264^ ^]^

Future research should focus on overcoming current limitations, such as the scalability of devices, the identification of universal rejuvenation factors, the need for specialized microfabrication techniques, which may not be accessible to all aging research laboratories, and the integration of microfluidics with advanced genetic and epigenetic and AI‐driven tools. By addressing these challenges, we can move closer to realizing the full potential of these technologies in promoting human health and longevity.

## Conflict of Interest

J.P.M. is CSO of YouthBio Therapeutics, an advisor/consultant for the BOLD Longevity Growth Fund and NOVOS, and the founder of Magellan Science Ltd, a company providing consulting services in longevity science. E.M. is the founder of BioRecode Ltd. R.D.K. is the co‐founder of AIM Biotech, a company that manufactures microfluidic platforms. He also receives research support from the following companies: AbbVie, Amgen, Boehringer‐Ingelheim, Daichi‐Sankyo, Eisai, EMD‐Serono, Novartis Takeda, The other authors declare no conflict of interest.

## Author Contributions

L.Z.‐D. and E.M. conceived, designed, and drafted the manuscript. V.J. contributed to the writing, design, and creation of graphical illustrations. G.M.C., R.D.K., and J.P.M. contributed to drafting, oversight, and substantial revisions. All authors reviewed, commented on, and approved the final manuscript.
